# Workplace violence and gender discrimination in Rwanda's health workforce: Increasing safety and gender equality

**DOI:** 10.1186/1478-4491-9-19

**Published:** 2011-07-19

**Authors:** Constance J Newman, Daniel H de Vries, Jeanne d'Arc Kanakuze, Gerard Ngendahimana

**Affiliations:** 1 IntraHealth International, 6340 Quadrangle Dr. Suite 200, Chapel Hill, North Carolina, 27517, USA; 2 University of Amsterdam, Amsterdam Institute for Social Science Research, Amsterdam, the Netherlands; 3 Public Service Commission, P.O. Box 6913, Kigali, Rwanda; 4 USAID HIV/AIDS Clinical Services Program--Northern Zone, BP 6199 Centenary House, Parcel #16 4th floor, Kigali, Rwanda

## Abstract

**Background:**

Workplace violence has been documented in all sectors, but female-dominated sectors such as health and social services are at particular risk. In 2007-2008, IntraHealth International assisted the Rwanda Ministries of Public Service and Labor and Health to study workplace violence in Rwanda's health sector. This article reexamines a set of study findings that directly relate to the influence of gender on workplace violence, synthesizes these findings with other research from Rwanda, and examines the subsequent impact of the study on Rwanda's policy environment.

**Methods:**

Fifteen out of 30 districts were selected at random. Forty-four facilities at all levels were randomly selected in these districts. From these facilities, 297 health workers were selected at random, of whom 205 were women and 92 were men. Researchers used a utilization-focused approach and administered health worker survey, facility audits, key informant and health facility manager interviews and focus groups to collect data in 2007. After the study was disseminated in 2008, stakeholder recommendations were documented and three versions of the labor law were reviewed to assess study impact.

**Results:**

Thirty-nine percent of health workers had experienced some form of workplace violence in year prior to the study. The study identified gender-related patterns of perpetration, victimization and reactions to violence. Negative stereotypes of women, discrimination based on pregnancy, maternity and family responsibilities and the 'glass ceiling' affected female health workers' experiences and career paths and contributed to a context of violence. Gender equality lowered the odds of health workers experiencing violence. Rwandan stakeholders used study results to formulate recommendations to address workplace violence gender discrimination through policy reform and programs.

**Conclusions:**

Gender inequality influences workplace violence. Addressing gender discrimination and violence simultaneously should be a priority in workplace violence research, workforce policies, strategies, laws and human resources management training. This will go a long way in making workplaces safer and fairer for the health workforce. This is likely to improve workforce productivity and retention and the enjoyment of human rights at work. Finally, studies that involve stakeholders throughout the research process are likely to improve the utilization of results and policy impact.

## Background

Workplace violence--which includes physical assault, verbal abuse, sexual or racial harassment, bullying or mobbing--affects occupational health worldwide. In 2002, the International Labour Organization, International Council of Nurses, World Health Organization, and Public Services International (ILO/ICN/WHO/PSI) Joint Programme on Violence in the Health Sector defined workplace violence as "Incidents where staff are abused, threatened or assaulted in circumstances related to their work, including commuting to and from work, involving an explicit or implicit challenge to their safety, well-being or health" [1]. The publication of this report officially brought the issue to the attention of public health researchers and practitioners [2]. This and other contemporaneous studies demonstrated the various effects and consequences of violence at the individual, organizational, and societal levels, noting that such incidents disrupt fundamental freedoms and rights in the workplace and often lead to depression, anxiety, physical disability, resignation, dismissal, transfer, absenteeism, lowered quality of care, decreased workplace productivity, increased costs to health systems, or even death [3].

In 2007-2008, IntraHealth International collaborated with the Rwandan Ministries of Health, Public Service and Labour, Gender and Family Protection, and Justice, as well as the Rwanda Health Workers union and other stakeholders, to conduct a study of workplace violence in the Rwandan health sector [4]. The study yielded findings about the relative importance of individual, organizational and societal factors that contributed to the emergence of workplace violence, and highlighted existing policies and programs that might be expanded to respond to the prevalent violence. The study report did not include information on the actions agreed upon to address the situation by each of the study stakeholders.

In this article, the authors analyze a subset of those study data to examine gender patterns of violence perpetration and victimization in conjunction with other data linking gender and workplace violence. Further, the authors examine other gender-related research from Rwanda to illuminate the connections between societal violence against women and workplace violence. Finally, the authors document stakeholders' recommendations for action and examine the effects that the study findings had on the policy environment within Rwanda, in order to offer suggestions that may assist human resources (HR) leaders and managers to improve the working conditions for and the productivity and retention of the health workforce.

### Relevant literature

Incidents of violence in the workplace occur in all sectors, but employees in female-dominated sectors such as health and social services are particularly vulnerable [5][6]. Experts in the field acknowledge the role of gender and apparently consider acts of workplace violence to be "gender-based," i.e. directed specifically against a woman because she is a woman, derived from unequal power relationship, or affecting women disproportionately [7]. For example, a fact sheet from the International Council on Nursing (ICN) noted that "[n]inety-five percent of nurses around the world are women. Attitudes towards women are often reflected in interactions with the profession," and particularly, that women were subjected to both domestic and workplace violence [8]. This makes explicit the link between violence experienced at home and at work. The ILO/ICN/WHO/PSI Joint Programme Report pointed out that, while both men and women seemed to be at risk, women were the victims in the majority of cases of workplace violence simply because the majority of workers in the health sector are women [9].

At the time of the Rwanda study, the researchers had difficulty finding research on workplace violence that linked gender-based violence or gender discrimination to workplace violence (beyond sexual harassment). Most works touched only the surface of a deeper relationship between gender and violence in the health workplace [10]. Baines' 2006 qualitative research, an exception to this, explored the link between women's vulnerability to violence and the gendered division of labor, where women are concentrated in tasks with greater risk of exposure to violence--such as social service workers who must provide intimate care and support to clients who are angry, frightened or in distress. Baines' results revealed that male social service workers were insulated from violence and stress by the types of tasks they took on (e.g. recreation rather than counseling), that violence against female workers was sexualized, and that clients appeared to know whom they could and could not attack with impunity. Like the ICN, Baines' study also drew parallels between the gender dynamics of professional care-giving and intimate partner violence [11].

The foregoing work suggested that a cause of workplace violence might be gender-based violence or discrimination in the larger society. As of 2007, there had been no human resources (HR) assessment in Rwanda, and little was known about the health workers' experience of workplace violence or any form of discrimination, including gender discrimination (defined as "[a]ny distinction, exclusion or restriction made on the basis of socially constructed gender roles and norms that prevents a person from enjoying full human rights" [12]). However, there existed gender policy guidance in Rwanda and some relevant local research on gender-based violence. The original impetus for the study was found in Rwanda's 2004 Gender Policy, which acknowledged that sexual harassment was a problem for girls in school and that it could nevertheless constitute a significant form of oppression and discrimination for Rwandan women at work [13]. In addition, there was research evidence suggesting that violence against women in Rwanda was a societal problem that might find its way into health workplaces. For example, the 2005 Rwanda Demographic and Health Survey found high levels of physical and sexual violence against women in the population [14]. There were also results from local studies demonstrating that domestic workers in Rwanda had experienced physical battery, sexual and emotional, economic deprivation and various forms of marginalization [15], and that expectant mothers attending Prevention of Mother to Child Transmission (PMTCT) services had experienced several forms of violence, but did not disclose it because of cultural norms of discretion, secrecy, modesty and fatalism in the face of violence (especially regarding sexual violence). These PMTCT clients also mentioned fear of retaliation by the perpetrator and of failure to act by law enforcement as reasons for remaining silent [16]. A study conducted by Rwanda's Ministry of Gender and Family Promotion in 2004 documented that over a third of women interviewed had been targets of "obscene discourse" in public places, including workplaces [17]. Rwandan proverbs also provide insight into the value placed on women's docility, the lack of weight carried by women's opinion, and the role of harshness and violence in controlling women's behavior [18]. Violence against women in Rwanda seems to have been enshrined in traditional sayings and buttressed by *de facto *and *de jure *discrimination in all spheres of life (though Rwanda now may be said to have put in place strong laws and policies to promote gender equality [19][20][21]).

### Study rationale

If organizational culture "neither erases nor diminishes national culture" [22], then it seemed reasonable to suppose that violence and discrimination occurring in the larger Rwandan society might manifest itself at work. It has been contended that discrimination can lead to the targeting or increased vulnerability to violence of girls and women [23] and that both problems should be addressed simultaneously [24], but the link between gender discrimination and workplace violence in the health sector needed further delineation through research. A better understanding of gender discrimination in health workplaces and its linkages to violence could hold promise for making working conditions safer and more equitable for Rwanda's largely female health workforce. More specificity would help HR leaders and stakeholders develop appropriate, proactive, and targeted policies and programs to increase workplace safety, security, and gender equity. Rwanda's Gender Policy and gender-based violence (GBV)-related research evidence indicated a need for workplace research. On the basis of this, IntraHealth International assisted Rwanda's Ministries of Health, Public Affairs and Labor, Gender, and the Health Workers' Union to explore and respond to violence in health workplaces.

## Methods

Formative research was conducted in early 2007 and consisted of interviews with policy makers, a focus group with health personnel, and a review of national labor and gender policies to assess interest and the extent to which various forms of violence were recognized in Rwanda. This formative research informed the development of data collection instruments; the identification of avenues of data analysis; and the generation of culturally appropriate descriptions of workplace violence and gender discrimination, including associated behaviors.

Data collection for the study took place in July 2007, and combined qualitative and quantitative approaches to determine the prevalence of workplace violence and its forms, victims, and perpetrators; identify contributing factors to workplace violence, including gender-related factors; describe victims' reactions and consequences; and describe any existing workplace violence policy and programs that could be reinforced or extended to address the issue. The study made use of six data collection tools: a health workers survey, facility manager and key informant interviews, patient focus groups and a facility risk assessment inventory (NB: This article draws only from a subset of health worker survey, key informant and facility manager interview, and facility audit results, in addition to information collected following dissemination of the study report). The forms of violence studied were verbal abuse, bullying, physical attack and sexual harassment. Exploration of the influence of gender on workplace violence focused on individual, organizational, facility-specific and societal factors contributing to workplace violence. The health workers survey included open and closed-ended questions covering forms of gender discrimination not measured in previous studies of workplace violence (e.g. workers' self report on equal access to jobs, training and career advancement; equal treatment of men and women; pregnancy and family responsibility discrimination; the "glass ceiling" or vertical segregation; task segregation; and perceptions of women and men at work).

After the study report results were disseminated, the researchers conducted new analyses of health worker survey data to better understand the perpetrator/victim dyad, documented the recommendations made by the Rwandan study stakeholder institutions and reviewed the content of three versions of the national code regulating labor in Rwanda, to identify any policy impact the study may have had.

### Sampling

The health workers survey was carried out in fifteen of Rwanda's 30 administrative districts, which were selected at random. Within each district, three health facilities were then selected at random. The facility sample included referral hospitals, district hospitals, health centers, clinics, and public health units or health posts, each of which were managed either by the government (public) or by non-governmental organizations authorized by the government (accredited facilities) or, in some cases, by the private sector. The health worker sample consisted of those who were in the randomly selected facilities on the day data collectors arrived at the targeted sites. Wherever possible, female and male health workers were selected to reflect the proportion of men and women believed to be in the population of Rwandan health workers (i.e. seven female and three male, yielding a stratified sample). A total of 297 health workers were surveyed; 205 were women and 92 men. Of the total number, 158 were from urban sites and 139 from rural sites. Among the 44 health facilities' directors, 20 were selected at random to be interviewed. When the director was not available, his/her assistant was interviewed. Table 1 shows the distribution of health cadres in the sample.

**Table 1:** Distribution of cadres in the sample

Cadre	Frequency	%
Physician	12	4.0

Auxiliary nurse	29	10

Nurse	157	53

Midwife	14	5

Technician	45	15

Social worker	40	14

**Total**	**297**	**100.0**

The researchers faced some challenges in documenting gender discrimination. First, de facto discrimination may exist, but may not be perceived or admitted if official policy and public rhetoric strongly promote gender equality and nondiscrimination (as they do in Rwanda), or when discrimination--like violence--is normalized. Second, de facto gender discrimination may exist, but may not be appraised because subjects of discrimination may lack direct evidence. An example of this was seen in the case of vertical segregation in top health facility management, where study respondents did not have access to objective data on the extent of male overrepresentation in the highest health management tier. Third, discrimination may be perceived but hard to substantiate because it is not always possible to access administrative records that contain objective, gender-disaggregated data on training and promotion opportunities or salary. Because of this, future researchers should, whenever possible, try to capture manifestations of gender discrimination and violence through both qualitative and quantitative means. This is not meant to discredit self-reports of discrimination experienced by health workers. However, in some cases it might be possible to link perceptions of discrimination to objective measures such as gender disaggregated data on salary, training attendance or promotions, gender composition of top management positions, or analysis of the allocation of work tasks and workloads.

### Data analysis

Qualitative data were analyzed for content and trends. Survey, interview, and facility data were collated by Rwandan consultants in a database using Excel. Generally, quantitative data were analyzed using a basic statistical package for cross-tabulation of the various prevalence types by variables of interest (such as 'equal treatment at work' and 'violence'), and conducting chi-square analyses to determine if distributions deviated significantly from the expected (alpha below 0.05). Trends were further analyzed using bivariate correlation analyses. Logistic regression was used to relate the chance of something happening (i.e. workplace violence) to a set of associated factors (e.g. sex, education or cadre of health worker, location or sector of facility, facility security, culture of respect, gender equality), allowing the researchers to assess the strength of the association of the various factors with the chance of workplace violence being experienced. Based on exploration of variables which showed trends toward significance, a logistic regression model was created with the variable "experience of violence" as dependent variable with no-prevalence as the reference category and eleven independent variables. A significant overall model (Chi2(33)-99.95,*P *= 0.00) was found which explained 25% of the variation (Pseudo R-sq = 0.25) and which had an adequate fit. The odds ratio was calculated to determine the chances of workplace violence being experienced. When the odds were above one, the variable increased the odds of violence relative to a variable. Below one, the odds declined relative to a variable. Finally, some graphics may include a denominator that is not the same as the sample size of 297, which indicates the number of responses to that question.

### Limitations

The planned sample size of 450 was not achieved, because the data available for health worker postings were not up to date, and because of health worker unavailability on the day of the survey, resulting in a smaller sample of 297 survey respondents. About 20% of those sampled were either line managers or senior managers (i.e. facility director), with 3% of the total being senior managers. This could potentially impact the results, but the research team nevertheless kept these staff in to have a sample size big enough for statistical analysis. However, since the senior managers comprised so small a percentage, their influence on the trends is relatively small. Because the data were based on a retrospective survey of self-ascribed respondent behavior over the past year, findings should be taken with some caution, as a response bias is likely to be of some influence. To minimize response bias, culturally appropriate tools in the local language were used and data were obtained by experienced and trained data collectors. The process of informed consent was rigorously followed for each respondent and, if needed, referrals were provided to psychological support services.

### Stakeholder processes

Studies have shown that those in charge of making policy-related decisions rarely use evaluation findings as the basis for those decisions, and this is likely to apply to research findings as well. In light of this, the research team applied principles of utilization-focused evaluation [25], to increase the relevance of study results for the primary users and application in subsequent HR policy-making and planning. The approach involved wide consultative processes from the study's inception to find out what various stakeholders thought was worth knowing about workplace violence. The researchers then worked over two years with a steering committee comprised of "focal points" from primary stakeholder institutions such as the Rwanda Ministries of Health, Public Service and Labour, Gender and Women's Promotion, Justice, and the Rwanda Health Workers' Union. The institutional focal points met at various stages to review the research proposal and tools; provide input on data sources; identify their data analysis priorities; assist in interpretation of results; and identify their institutions' future roles in dissemination and use of results. Stakeholder priorities were key to the content of the tools and data analysis. These priorities directed the questions; for example, questions about the victim's marital status and the impact of violence on families were included in the health workers survey tool, while other questions or response options were not. The research also included activities to build focal points' technical capacity, provide opportunities to engage in policy dialogue, plan stakeholder institutions' internal briefings and prepare presentations for a national multisectoral results dissemination workshop, which consisted of concrete institutional recommendations to reduce workplace violence in the health sector.

## Results

### Prevalence

Violence was a real problem in Rwandan health workplaces and was perceived as such. Approximately 39% of health workers reported experiencing at least one form of workplace violence in the twelve months prior to the study: 27% of respondents had experienced verbal abuse, 16% were bullied, 7% encountered sexual harassment and 4% were physically assaulted. Verbal abuse was the most prevalent form, and physical violence the least prevalent. Sexual harassment was not the most prevalent form but it was the most frequent for those who experienced it. The prevalence of psychological violence (including bullying, verbal abuse and sexual harassment) was greater than that of physical assault.

### Patterns of perpetration and victimization

**Figure 1:**
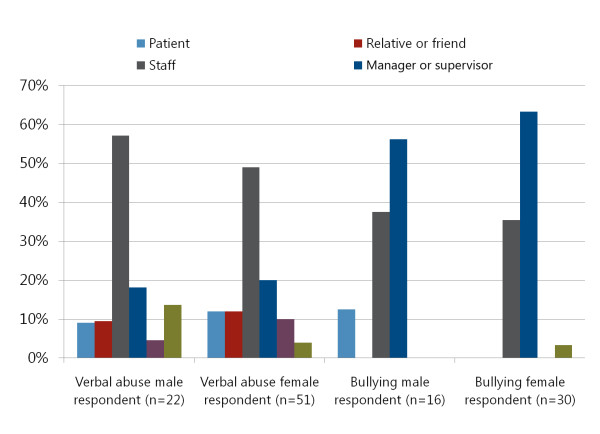
**Type of perpetrator of verbal abuse and bullying by sex of respondent (n = 119)**.

Health worker survey data on the relationship of perpetrator to victim by type of perpetrator and type of violence were sufficiently robust to demonstrate perpetrator-victim patterns, especially for verbal abuse and bullying. Survey respondents were asked what type of perpetrator was involved in the last instance of violence experienced. Figure 1 shows these data, where male respondents who answered this question reported that they were verbally abused most by other staff, manager/supervisors, and then by an unidentified "other" category. Female respondents likewise reported being verbally abused most by other staff and managers/supervisors, followed by members of the general public. Managers/supervisors and other staff were implicated most often in bullying, though managers/supervisors were far more often reported to have perpetrated bullying. Managers/supervisors appeared to bully female respondents more than male respondents (however, while these observed patterns are meaningful, there were not statistically significant using a Pearson chi-square test).

Respondents of the health workers survey were also asked the following multiple choice question for each type of violence (noting that a respondent possibly could choose multiple types of violence): *"Please think of the last time you experienced (X form of) violence at work. 1) How many people perpetrated the violence? 2) Was (were) the perpetrator(s): a) male; b) female; or c) both?*" Table 2 shows instances of perpetration by type of workplace violence and sex of perpetrator (or perpetrators) and victim. The percentages in Table 2 represent the perpetrator(s) identified at the last instance of violence which was experienced, for the four types of violence, giving an idea of the frequency of the types of violence experienced by the sample of health workers.

**Table 2:** Instances of perpetration by type of workplace violence and sex of victim

Perpetrator identified as:	Verbal abuse experienced by		Bullying experienced by		Sexual harassment		Physical attack experienced by experienced by		
	Male victim	Female victim	Male victim	Female victim	Male victim	Female victim	Male victim	Female victim	Total
Male	26%	20%	69%	48%	0.0%	88%	50.0%	57%	**58**

Female	58%	54%	19%	36%	80.0%	0.0%	25.0%	43%	**55**

Both male and female	16%	27%	13%	16%	20.0%	13%	25.0%	0.0%	**25**

**Total (100%)**	**19**	**41**	**16**	**31**	**5**	**15**	**4**	**7**	**138**

Tables 3, 4, 5 and 6 have been broken out to illustrate patterns of perpetration and victimization for each form of violence.

**Table 3:** Patterns of perpetration and victimization for verbal abuse

	Male victim	Female victim	Total
**Male perpetrator**	(5) 26%	(8) 20%	(13) 22%

**Female perpetrator**	(11) 58%	(22) 54%	(33) 55%

**Male and female**	(3) 16%	(11) 27%	(14) 23%

**Total**	(19) 32%	(41) 68%	60

**Table 4:** Patterns of perpetration and victimization for bullying

	Male victim	Female victim	Total
**Male perpetrator**	(11) 69%	(15) 48%	(26) 55%

**Female perpetrator**	(3) 19%	(11) 35%	(14) 30%

**Male and female**	(2) 13%	(5) 16%	(7) 15%

**Total**	(16) 34%	(31) 66%	47

**Table 5:** Patterns of perpetration and victimization for sexual harassment

	Male victim	Female victim	Total
**Male perpetrator**	(0) 0%	(13) 87%	(13) 65%

**Female perpetrator**	(4) 80%	(0) 0%	(4) 20%

**Male and female**	(1) 20%	(2) 13%	(3) 15%

**Total**	(5) 25%	(15) 75%	20

**Table 6:** Patterns of perpetration and victimization for physical attack

	Male victim	Female victim	Total
**Male perpetrator**	(2) 50%	(4) 57%	(6) 55%

**Female perpetrator**	(1) 25%	(3) 43%	(4) 36%

**Male and female**	(1) 25%	(0) 1%	(1) 9%

**Total**	(4) 36%	(7) 64%	11

### Patterns of perpetration and victimization for verbal abuse (Table 3)

In response to the survey question, *"Please think of the last time (instance) you were verbally abused at work,*" respondents said that they had been verbally abused by a man in 22% of the instances and by a woman in 55% of the instances. Respondents reported that that they had been verbally abused by both men and women in 23% of the instances. Both male and female health workers were more like to be verbally abused by women, at 58% and 54% respectively, with the exception that female victims were more likely to be abused by men and women together. Slightly more than two thirds (68%) of the victims were female health workers, and slightly under one third (32%) were male health workers, which mirror their proportions in the stratified sample.

### Patterns of perpetration and victimization for bullying (Table 4)

In response to the survey question, *"Please think of the last time you were bullied at work," *health workers reported that they had been bullied by a man in 55% of the instances and by a woman in 30% of the instances, with the remainder bullied by both. A sizeable majority of male health workers reported they had been bullied by a man (69%) and slightly less than one fifth (19%) had been bullied by a woman at the last instance of bullying. In somewhat less than half (48%) of the last instances of bullying, female health workers reported that the bully was a man, and slightly more than a third (35%) reported the bully had been a woman. Female health workers also reported they had been bullied by both men and women in 16% of the last instances of bullying. Two thirds (66%) of the victims were female health workers and one third (34%) of the victims were male health workers at the last instance of bullying. In terms of a pattern of bullying, we see that men perpetrated bullying in most instances and that the bullying was experienced by men and women according to their proportion in the study sample.

### Patterns of perpetration and victimization for sexual harassment (Table 5)

In response to the survey question, *"Please think of the last time you were sexually harassed at work," *health workers reported that they had been sexually harassed by a man in 65% of the instances, by a woman in 20% of the instances, and by both in 15% of the instances. Of the health workers who reported being sexually harassed, one quarter (25%) were men and three quarters (75%) were women, which does not mirror the proportions in the sample. This means that female health workers in this sample were disproportionately victims of sexual harassment. There was a strong pattern for the perpetrator to be the opposite sex of the victim.

### Patterns of perpetration and victimization for physical attack (Table 6)

In response to the survey question, *"Please think of the last time you were physically assaulted/attacked at work," *we see (even though the overall number of instances is only eleven) that health workers reported that they had been physically attacked by a man in 55% of the last instances, and by a woman in 36% of the instances. Male health workers were more likely to be physically attacked by a man than otherwise; while female health workers were almost equally likely to be physically attacked by either a man or a woman, though slightly more by a man. At the last instance of physical attack, close to two thirds (64%) of the victims were female health workers, and slightly more than one third (36%) of the victims were male health workers, in keeping with the sample proportions.

### Summary

In this study, gender appeared to be implicated in patterns of perpetration and victimization. Taken together, the Tables 3 through 6 suggest that men were mentioned as perpetrators in the most instances of bullying, physical attack, and sexual harassment, while women were more likely to be mentioned as perpetrators in most instances of verbal abuse. Male and female health workers were equally victims of verbal abuse, bullying, and physical attack. Only in sexual harassment did the proportion of male and female victims *not *reflect their proportions in the stratified sample, making sexual harassment the form of violence of which female health workers were disproportionately the victims. Female health workers were also more likely to be verbally abused by both women and men. In the pattern of bullying, male health workers were much more likely to be victimized by men. With respect to physical attack, female health workers were more likely to be attacked by a woman or a man, whereas male health workers were more likely to be attacked by men (although the number of reports was small).

### Reactions to workplace violence

Table 7 displays health workers' reactions to the experience of workplace violence. In response to the question *"Have you ever left, or considered leaving the health care sector, or this job, due to your experience of (x form of) violence?" *most respondents reported that they either did nothing or only considered leaving their (health sector) job. Only 4% of male workers reported that they had ever actually quit a job as a result of verbal abuse. Of female workers, 10% and 7% reported that they had ever actually left a job because of bullying and sexual harassment, respectively. These findings suggest that workplace violence is an occurrence that most health workers may feel they can or must live with, almost as a normal part of the job, but that the experiences of bullying and sexual harassment are more likely to result in a female health worker leaving a job.

**Table 7:** Reactions to workplace violence

Reaction	Male HCWs (N = 48)				Female HCWs (N = 111)			
	Verbal abuse (n = 24)	Physical violence (m = 4)	Bullying (n = 16)	Sexual harass-ment (n = 4)	Verbal abuse (n = 36)	Physical violence (n = 7)	Bullying (n = 31)	Sexual harass-ment (n = 17)
Did nothing	61%	40%	50%	80%	50%	83%	39%	47%

Considered leaving	35%	60%	50%	20%	46%	17%	52%	47%

Left the job	4%	0%	0%	0%	4%	0%	10%	7%

The health worker survey data also showed that health workers' disclosure of incidents varied with the type of violence. In most cases--especially those of verbal abuse, bullying, and physical violence--subjects disclosed the incident to colleagues, friends and family. However, in 40% of sexual harassment cases, the victim did not disclose the occurrence to anyone. This was not surprising given the norms of silence around sexual violence in Rwanda. Fewer than 20% of victims of most forms of violence reported the incident to their supervisor. This low overall reportage rate is also not surprising, given that hierarchical superiors (i.e. managers orsupervisors) were reportedly involved in the perpetration of all types of violence (and especially bullying, see Figure 1). When asked if the supervisor or the director of the facility took measures to help the victim or to respond to violence, only 30% of health workers agreed. These findings become more important in conjunction with the health workers' self-reports of feeling a loss of dignity, trauma, lower productivity and actual absenteeism following the experience of violence.

### Gender discrimination and workplace violence

In this section, we examine the influence of gender discrimination and violence. To what extent is gender discrimination a feature of, or a context in which, violence occurs? The qualitative and quantitative data from the health workers survey and key informant interviews suggested that female health workers experienced problems at work which are recognized forms of gender discrimination and that these problems co-occurred with violence.

### Discrimination based on pregnancy and family responsibilities

The researchers asked health workers if they agreed or disagreed that various forms of discrimination occurred at work. Forty-nine per cent of (male and female) respondents agreed that women did not encounter the same problems as men at work, and 41% agreed that women were more exposed to violence. Sixty-eight per cent of respondents pointed to pregnancy, childbirth, and family and child care responsibilities as factors that prevented women from fully participating at work. Qualitative data from the health workers' survey also suggested that female workers perceived their career progress to have been adversely affected by the unique problems faced by women at work. Some female workers reported that performance evaluations or opportunities for promotion had been influenced by their maternity status; others reported that they had been demoted after pregnancy without cause, or were not hired for a position because of presumed future pregnancy. One respondent noted that "When I had not yet delivered, I was deputy director; after delivery, I was demoted for no reason but I think it was because of my pregnancy."

### Occupational segregation

The study revealed vertical segregation of the facility director's job by sex (i.e. the 'glass ceiling'). Survey data were analyzed with attention to the number of men and women who occupied the top management (facility director) jobs. While only 16% of health workers in the survey sample believed that women did not have the same chance as men of being hired for jobs for which they are qualified, women actually *did not *appear in the top management jobs at the same rates as men. Even though men made up only about 31% of the sample, they constituted 60% of directors in the sample facilities. These findings suggested that vertical segregation (a form of discrimination) may exist but is not necessarily perceived. However, about one out of four service providers agreed that task assignments for male and female workers occupying the same job differed either in types or volume, suggesting some (horizontal) gender segregation of tasks that was observable to respondents. As a female study participant noted, "Some people seem to think that certain activities are reserved only for men."

### Negative stereotypes of female workers

Sexual harassment, problems at work related to pregnancy and family responsibilities, and occupational segregation co-occurred with negative stereotypes about female health workers, such as an unwillingness to speak up, weakness, indecisiveness and incompetence. One survey respondent observed that women at work "just don't know how to make decisions in a sure and certain way." Another noted that women "are not even capable of pulling out a tooth." These negative stereotypes may be the foundation on which violence (as well as other forms of workplace discrimination) rest. Indeed, according to a key informant, beliefs about Rwandan women in the health workplace rationalize violence: "There is a tendency to say that women are weak in the broadest sense... and, in some cases, the violence that women are subjected to stems from this situation."

### Gender equality and reduced odds of workplace violence

In this section, we consider the relationship between gender equality in the workplace as one of several variables influencing health workers' experience of violence. The study considered several types of factors as possible contributors to workplace violence: individual factors (e.g. sex, age, marital status, seniority, education); general factors (e.g. sector, facility location, staff workload); facility security; and behavioral factors (e.g. a culture of respect and gender equality).

Table 8 shows that, after the general factor of facility sector and location and the facility security factor of building entrance visibility, the most important contributing factors to the experience of violence in Rwandan health workplaces were gender inequality and the lack of a culture of mutual respect. Rather than the characteristics of individual workers (such as age or seniority), and after sector and geographical location and visibility of a facility entry, gender equality comes to the fore: when men and women have an equal chance to get hired for jobs for which they are qualified, the odds of violence were calculated to be about *six times lower *(at 0.2), compared to a setting where there is no equal chance. Further, when men and women receive equal treatment at work, the odds of workplace violence were about *five times lower *(at 0.2), compared to a setting in which there is no equal treatment. The culture of respect variables are of lesser importance compared to the gender equality variables, though respect between supervisors and staff also especially lowers workplace violence. The gender equality variables are thus considered as the more influential behavioral contributor to reduced workplace violence.

**Table 8:** Logistic regression result: odds of experiencing violence

Variable	Variables of significance in the aggregated model of violence both at work and during travel to and from work	Sign. (p)	Odds Ratio (Exp(B))	***Inverse of Odds Ratio (if decline ) ***
**General **(Sector and location)	Religious sector (compared to Government)	.017	4.7	
	
	City (compared to Rural)	.012	11.8	
	
	Southern Province (compared to the North)	.002	0.1	(11)
	
	Kigali City (compared to North)	.009	0.1	(14)

**Facility security**	People loitering in neighborhood adjacent to or surrounding facility	.099*	2.7	
	
	Signs posted to indicate staff-only break areas (as compared to no signs)	.043	2.7	
	
	Building entrance is visible from the street and free of heavy shrub growth (as compared to entrance not being visible)	.002	0.1	(12)

**Culture of respect**	High level of perceived respect which patients show to staff at workplace (compared to low level of respect )	.066*	0.7	(1.4)
	
	High level of perceived respect that supervisors and staff show to each other at the workplace (compared to low level of respect)	.028	0.6	(2)

**Gender equality**	Perceived equal chance for men and women to get hired for jobs for which they are qualified in the health sector (as compared to unequal chance)	.009	0.2	(6)
	
	Perception of equal treatment at work received by men and women (as compared to unequal treatment)	.017	0.2	(5)

*Not highly significant

**Figure 2:**
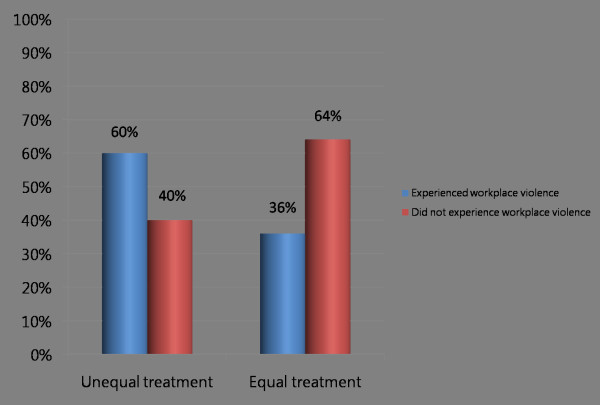
Do men and women receive equal treatment at work?

Figure 2 graphically depicts that perceived equal treatment at work was associated with a reduced percentage of health workers experiencing workplace violence.

Sixty per cent of respondents who perceived unequal treatment also indicated that they had experienced some form of workplace violence, compared to 36% of those who perceived equal treatment (Pearson chi2(1) = 9.388, P = 0.002).

**Figure 3:**
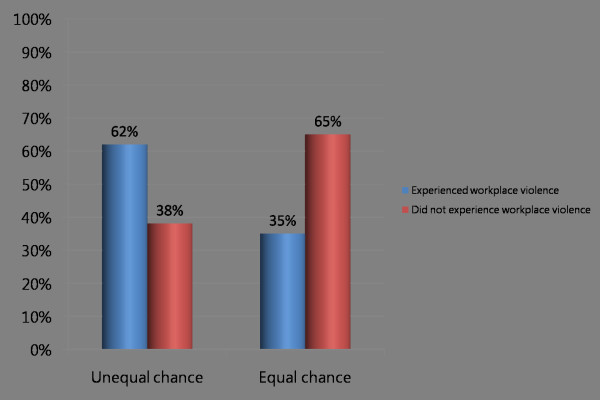
Do men and women have an equal chance to get hired for jobs for which they are qualified?

Further, Figure 3 shows that the perception of an equal chance to get hired for jobs for which the worker is qualified is associated with a reduction in the percentage of health workers experiencing workplace violence. Of respondents who perceived inequalities in the hiring process, 62% indicated that they had experienced some form of workplace violence, contrasted with 35% of respondents who had perceived equal chances for both men and women (Pearson chi2(1) = 11.639, P = 0.001).

### Stakeholders' use of results and study impact

The participatory, capacity-building approach oriented to relevance of results for stakeholders appeared to increase use of the study results and the impact of the study. Table 9 shows the actions recommended by focal points from the Rwandan stakeholder institutions, which demonstrated a desire for a multilevel, multisectoral response to violence in the health sector, as well as awareness of the need to address gender discrimination and violence together.

**Table 9:** Multilevel, multisectoral actions recommended to eliminate workplace violence and discrimination in Rwanda's health sector

By the Ministry of Health	By the Ministry of Labor	By the Ministry of Gender	By the Rwanda Health Workers Union
• Conduct a study on pregnancy discrimination	• Disseminate the Labor Law	• Train men and women to disclose violence	• Document and translate relevant texts into Kinyarwanda
• Develop and enact a health sector policy on workplace violence	• Develop a labor sector policy on worker safety and security	• Share information about, monitor and evaluate workplace violence	• Conduct information, sensitization and training campaign on legal texts
• Develop and enact a program to fight violence in health workplaces	• Build the capacity of labor inspectors	**Recommendations to the Ministry of Health:**	• Disseminate and enforce ILO standards on maternity protection and workers with family responsibilities
• Develop partnerships with MOH, MOL, MOG, Police, donors.	• Diffuse information on labor standards	• Train health workers on ethical behavior	• Develop a system to manage cases
	• Put in place workplace safety and security policies that address discrimination.	• Establish Ethics Committee at health centers	• Develop networks with other unions and human rights groups
		• Sensitize and train all health workers on violence and sanctions.	• Conduct a study on human rights at health workplaces.

### Impact

The study appears to have had impact on the national policy environment in particular, having contributed to the revision of the national Law Regulating Labour in Rwanda and to plans to revise the national GBV policy. First, analysis of Rwanda's three labor laws between 2001, 2007 and 2009 showed an evolution in language, wherein no mention is made of violence in 2001 and 2007, but in the 2009 version (one year after the study results were nationally disseminated), there appears in the national labor law a definition of GBV, and the following Articles [26]:

"Section 3: Protection of workers against violence or harassment

Article 9: Prohibition of gender-based violence

Article 10: Resignation in case of violence

Article 11: Prohibition of punishment [for reporting violence]

Section 4: Prohibition of discrimination in work matters."

The study report, which was subsequently disseminated in other stakeholder meeting venues (e.g. to the Rwanda Medical Association and Nursing Council) was also one of three to be included as a resource for the Ministry of Gender and Family Promotion's revision of the 2010 GBV policy [27].

## Discussion

The study shed light on the types, reactions and contributing factors to, and consequences of, workplace violence. Workplace violence is a real phenomenon within the health sector in Rwanda with individual, organizational, and societal impacts experienced by 39% of the health workers sampled. Male and female staff experienced violence, and mostly did nothing or only thought of leaving the job after the experience, even though the experience of violence was personally traumatic, interpersonally disruptive and organizationally depleting in terms of self-reports of absenteeism and lower productivity.

The study identified ways gender appears to influence workplace violence. The first was in patterns of violence perpetration and victimization. Sexual harassment was a particular problem for female health workers. In light of results from studies on violence against women in Rwanda described earlier, a culture of silence and resignation *vis a vis *violence against women, and the finding about nondisclosure of sexual harassment, it is probable that the prevalence of sexual harassment in Rwandan workplaces is a low estimate. It should be noted, nevertheless, that female workers appeared more likely to leave a job because of bullying and sexual harassment experienced at work, a finding that should be of interest to human resource managers who want to retain female health workers.

The second way gender appears to influence violence in health workplaces is in the forms of discrimination faced by female health workers. Sexual harassment co-occurred with problems related to managing pregnancy, motherhood and work, and negative stereotyping of women at work, the latter perhaps rationalizing violence against women in Rwanda's health workplaces. The findings on the over-representation of men in top management jobs and gender-based task assignment point to occupational segregation, a ubiquitous form of gender discrimination that typically results from multiple and accumulating discriminations during childhood, schooling, and entry into a career. The fact that female health workers appear to have experienced more than one type of discrimination and bias suggests a systemic substratum of gender discrimination in the health workplace that also deserves serious attention from HR policy makers and managers, because discriminatory working conditions, along with the experience of violence, can weaken female health workers' ties to the workforce. The finding that male perpetrators are overwhelmingly implicated in bullying male health workers may qualify as a form of gender-based violence worthy of further study. The role of the manager/supervisor in bullying also suggests a need to address the use and abuse of power in HR manager training.

Most striking was the finding that gender equality lowers the odds of experiencing violence at work. Put another way, this means that gender inequality at work (as illustrated by unequal treatment and unequal access to jobs) increases the odds of violence.

The study results suggest that violence against female health workers in Rwandan health workplaces may come about from a "category bias" [28] in which violence against female health workers is but one component of the wider problem of gender discrimination that denies women the opportunity to exercise employment rights and economic freedoms based on their biological and social roles. The link between gender inequality and workplace violence bore out the contentions that discrimination contributes to violence and that both should be dealt with simultaneously when planning and supporting a largely female workforce. Even in cases where male and female workers both experience workplace violence, it might have a greater or different impact on female workers if gender bias or discrimination limits their options, including the freedom to disclose and seek redress [29].

Finally, the gender-sensitive, multisectoral recommendations made by the stakeholder institutions and recent changes in Rwanda's labor law to address workplace violence are promising steps towards a goal of making the health sector safer and more gender-equitable for its workforce. It is hoped that the elimination of sexual harassment and other forms of gender discrimination will figure in Rwanda's new (2010) draft of the national GBV policy.

## Conclusions

Gender discrimination and inequality contribute to violence against women in the health sector. Because the key factors contributing to the emergence of violence in Rwandan health workplaces were institutional and behavioral rather than individual, it is likely that improved HR policy--and management practices that enact and enforce improved policy--could decrease some of the risks for violence. First, workplace violence research and workforce assessments should routinely measure the extent of systemic gender discrimination at work. Second, eliminating gender inequality, bias, and specific forms of discrimination should be a special concern in the development of both HR and workplace violence policies and programs, including reporting systems. HR policies should protect women against violence as well as against losing seniority, promotion prospects, or a job as a result of pregnancy or caring for children and family members. Countries that are signatories to international consensus documents on gender equality, such as the International Labour Organization's (ILO's) four gender equality labor standards, already have a policy context for policy and legal reform (i.e. See Conventions 111, 100, 156 and 183) [30]. Sectoral strategies should challenge gender stereotypes, discriminatory behavior, and all forms of violence against women. Finally, HR managers should institute nondiscrimination and antiviolence policies in workplace guidelines and codes of conduct and should vigorously enforce them. Training HR managers to be good stewards of the power vested in them, to identify gender discrimination when it is occurring and to manage the risks of violence in the workplace all should be high on the list of HR management priority actions. Taking action on the foregoing will go a long way in making health systems safer and more gender-equitable for its workforce, especially in increasing the enjoyment of human rights at work. This is in turn may increase the productivity and retention of the health workforce. Finally, studies that involve stakeholders through the research process are likely to improve the utilization of results and policy impact.

## Abbreviations

GBV: Gender-Based Violence; HR: Human Resources; ILO: International Labour Organization; ICN: International Council for Nurses; MOH: Ministry of Health; PSI: Public Services International; PMTCT: Prevention of Mother to Child Transmission; WHO: World Health Organization.

## Competing interests

The authors declare that they have no competing interests.

## Authors' contributions

CN conducted the formative research, conceptualized the study and designed, oversaw or gave input to all aspects of methodology, data analysis, interpretation, dissemination, report writing and French to English translation. JK reviewed instruments, results and reports, oversaw data collection and coordinated dissemination. GN gave input into formative research, study design, gave policy guidance, and reviewed results and the report. DdV reviewed the tools, developed the data analysis and sampling plans, analyzed data, and reviewed drafts of the study report. All authors reviewed and approved the final manuscript.
